# Comparison of SARS-CoV-2 Antibody Response 4 Weeks After Homologous vs Heterologous Third Vaccine Dose in Kidney Transplant Recipients: A Randomized Clinical Trial

**DOI:** 10.1001/jamainternmed.2021.7372

**Published:** 2021-12-20

**Authors:** Roman Reindl-Schwaighofer, Andreas Heinzel, Manuel Mayrdorfer, Rhea Jabbour, Thomas M. Hofbauer, Anne Merrelaar, Michael Eder, Florina Regele, Konstantin Doberer, Paul Spechtl, Constantin Aschauer, Maximilian Koblischke, Christopher Paschen, Farsad Eskandary, Karin Hu, Barbara Öhler, Arshdeep Bhandal, Sabine Kleibenböck, Rahel I. Jagoditsch, Bianca Reiskopf, Florian Heger, Gregor Bond, Georg A. Böhmig, Robert Strassl, Lukas Weseslindtner, Alexander Indra, Judith H. Aberle, Michael Binder, Rainer Oberbauer

**Affiliations:** 1 Division of Nephrology and Dialysis, Department of Internal Medicine III, Medical University of Vienna, Vienna, Austria; 2 Division of Cardiology, Department of Internal Medicine II, Medical University of Vienna, Vienna, Austria; 3 Department of Emergency Medicine, Medical University of Vienna, Vienna, Austria; 4 Center for Virology, Medical University of Vienna, Vienna, Austria; 5 Institute for Medical Microbiology and Hygiene, Austrian Agency for Health and Food Safety, Vienna, Austria; 6 Division of Virology, Department of Laboratory Medicine, Medical University Vienna, Vienna, Austria; 7 Paracelsus Medical University of Salzburg, Salzburg, Austria; 8 Wiener Gesundheitsverbund, Vienna, Austria

## Abstract

**Importance:**

Fewer than 50% of kidney transplant recipients (KTRs) develop antibodies against the SARS-CoV-2 spike protein after 2 doses of an mRNA vaccine. Preliminary data suggest that a heterologous vaccination, combining mRNA and viral vector vaccines, may increase immunogenicity.

**Objective:**

To assess the effectiveness of a third dose of an mRNA vs a vector vaccine in KTRs who did not have antibodies against the SARS-CoV-2 spike protein after 2 doses of an mRNA vaccine.

**Design, Setting, and Participants:**

This was a single center, single-blinded, 1:1 randomized clinical trial of a third dose of vaccine against SARS-CoV-2, conducted from June 15 to August 16, 2021, in 201 KTRs who had not developed SARS-CoV-2 spike protein antibodies after 2 doses of an mRNA vaccine. Data analyses were performed from August 17 to August 31, 2021.

**Interventions:**

mRNA (BNT162b2 or mRNA-1273) or vector (Ad26COVS1) as a third dose of a SARS-CoV-2 vaccine.

**Main Outcomes and Measures:**

The primary study end point was seroconversion after 4 weeks (29-42 days) following the third vaccine dose. Secondary end points included neutralizing antibodies and T-cell response assessed by interferon-γ release assays (IGRA). In addition, the association of patient characteristics and vaccine response was assessed using logistic regression, and the reactogenicity of the vaccines was compared.

**Results:**

Among the study population of 197 kidney transplant recipients (mean [SD] age, 61.2 [12.4] years; 82 [42%] women), 39% developed SARS-CoV-2 antibodies after the third vaccine. There was no statistically significant difference between groups, with an antibody response rate of 35% and 42% for the mRNA and vector vaccines, respectively. Only 22% of seroconverted patients had neutralizing antibodies. Similarly, T-cell response assessed by IGRA was low with only 17 patients showing a positive response after the third vaccination. Receiving nontriple immunosuppression (odds ratio [OR], 3.59; 95% CI, 1.33-10.75), longer time after kidney transplant (OR, 1.44; 95% CI, 1.15-1.83, per doubling of years), and torque teno virus plasma levels (OR, 0.92; 95% CI, 0.88-0.96, per doubling of levels) were associated with vaccine response. The third dose of an mRNA vaccine was associated with a higher frequency of local pain at the injection site compared with the vector vaccine, while systemic symptoms were comparable between groups.

**Conclusions and Relevance:**

This randomized clinical trial found that 39% of KTRs without an immune response against SARS-CoV-2 after 2 doses of an mRNA vaccine developed antibodies against the SARS-CoV-2 spike protein 4 weeks after a third dose of an mRNA or a vector vaccine. The heterologous vaccination strategy with a vector-based vaccine was well tolerated and safe but not significantly better than the homologous mRNA-based strategy.

**Trial Registration:**

EudraCT Identifier: 2021-002927-39

## Key Points

**Question:**

Does a heterologous SARS-CoV-2 vaccination strategy with the vector vaccine Ad26COVS1 result in a higher rate of antibody response compared with a homologous third dose of mRNA vaccine (mRNA-1273 or BNT162b2) in kidney transplant recipients who did not develop SARS-CoV-2 antibodies after 2 doses of an mRNA vaccine?

**Findings:**

This randomized clinical trial found that a third dose of SARS-CoV-2 vaccine in 197 kidney transplant recipients without antibodies after 2 doses of an mRNA vaccine induced an antibody response in 35% of the homologous (mRNA) group vs 42% of the heterologous (vector) group, with no statistically significant difference.

**Meaning:**

The findings of this randomized clinical trial show that homologous and heterologous vaccination strategies for a third SARS-CoV-2 vaccine dose in kidney transplant recipients are comparable, with both mRNA and vector vaccines achieving seroconversion in more than one-third of kidney transplant recipients. However, given the high rate of nonresponders after the third dose, additional strategies to induce an immune response in kidney transplant recipients are urgently needed.

## Teaser

This randomized clinical trial evaluated the effectiveness of a third dose of mRNA-based vs a vector-based vaccine in 201 kidney transplant recipients with no SARS-CoV-2 antibodies after 2 prior doses of an mRNA vaccine.

## Introduction

Kidney transplant recipients (KTRs) are considered at high risk for severe COVID-19 disease, and therefore, were prioritized for early SARS-CoV-2 vaccination.^1,2^ Immune response to vaccination is reduced in immunosuppressed individuals, including KTRs.^3^ In the US and the European Union, 2 classes of SARS-CoV-2 vaccines are currently available: (1) mRNA (mRNA-1273 [Moderna] and BNT162b2 [PfizerBioNTech]) and (2) viral vector (ChAdOx1 [AstraZeneca] and Ad26COVS1 [Janssen]). Among these, Ad26COVS1 is the only vaccine that has been shown to induce a protective immune response after a single dose.^4^

Recent studies from Israel and the US confirmed that only 54% and 38% of KTRs developed antibodies against the spike protein following vaccination with 2 doses of an mRNA vaccine.^5,6^ The type of immunosuppression substantially affects the immune response in KTRs, and patients treated with co-stimulation blockade (belatacept) show even lower immune response rates (only 6%).^7,8^ Additional risk factors identified for nonresponsive KTRs are the time elapsed since the transplant and lymphocyte-depleting induction therapy.^9^

Proposed strategies to improve response to SARS-CoV-2 vaccination include additional dosing and heterologous booster vaccination. The T-cell response against the SARS-CoV-2 spike protein after mRNA vaccination is severely impaired in KTRs.^10^ Adenovirus-based vector vaccines introduce a stronger T-cell response in mouse models that could help overcome immunosuppression in KTRs.^11,12^ Recent data showed a higher reactogenicity of heterologous boosting among the general population, as well as higher antibody and T-cell responses after heterologous compared with homologous vaccination.^13,14,15^

Initial reports of a third dose of a SARS-CoV-2 vaccine in non- or low-responders showed promising results, with more than 1 in 3 patients developing antibodies against SARS-CoV-2.^16,17,18^ We hypothesized that in KTRs who did not develop antibodies against SARS-CoV-2 after 2 doses of mRNA vaccines, a heterologous vaccination strategy with a viral vector would improve the humoral response against SARS-CoV-2 compared with a third dose of the same mRNA vaccine.

## Methods

### Study Design

This was an investigator-initiated, single center, single-blinded, 1:1 randomized clinical trial to assess the effectiveness of a heterologous vaccination strategy using Ad26COVS1 (viral vector) compared with a homologous strategy using either BNT162b2 or mRNA-1273 (mRNA) as a third dose in KTRs who did not develop SARS-CoV-2 spike protein antibodies after 2 doses of an mRNA vaccine. Patients were blinded for the type of vaccine administered. The trial was conducted from June 15 to August 16, 2021, at the Department of Nephrology and Dialysis at the Medical University of Vienna (Austria). The trial was registered with the European Union Clinical Trial Register and was approved by the ethics committee of the Medical University of Vienna (No. 1612/2021) as well as by the Austrian Agency for Health and Food Safety. The complete trial protocol is available in Supplement 1. The study was conducted in accordance with the principles of the Declaration of Helsinki and was sponsored by the Medical University of Vienna. All study participants provided written informed consent before study entry.

### Study Cohort

The study population comprised adult KTRs without detectable SARS-CoV-2 spike protein antibodies 4 or more weeks after the second dose of an mRNA vaccine. Participants with a prior documented SARS-CoV-2 infection, a positive test result for SARS-CoV-2 nucleocapsid antibodies at the time of screening, or who were being treated with triple anticoagulation therapy were not eligible for study participation. A negative pregnancy test result was required for all female participants of reproductive age prior to third-dose vaccination.

### Outcomes

The primary outcome was the difference in seroconversion rate (ie, detectable SARS-CoV-2 spike protein antibodies at a level >0.8 units [U]/mL) between the mRNA and vector vaccination groups after 4 weeks (29-42 days) following the third dose. In secondary analysis, we used a higher cutoff level (>15 U/mL), which exhibits a positive predictive value of more than 99% for presence of neutralizing antibodies (per the immunoassay manufacturer’s instructions) and cutoff levels recently reported in the literature^19,20,21^ to correlate with neutralizing capacity, as well as the reduced risk for COVID-19 infection (>100 U/mL^19^; >141 binding antibody units [BAU]/mL^20^; >264 BAU/mL^21^). Note that BAU/mL were converted to U/mL (U/mL = 0.972 × BAU/mL). Additional secondary outcomes included differences in SARS-CoV-2 antibody levels between mRNA and vector vaccinated groups as well as interferon-γ produced by SARS-CoV-2 specific T-cells. Furthermore, differences in the amount of interferon-γ produced by SARS-CoV-2–specific T-cells after the second and third vaccination were evaluated.

We also explored patient characteristics at baseline and any potential associations between characteristics and vaccine response. Differences in reactogenicity were assessed using a visual analogue scale (0, no symptoms; 1-2, mild; 3-4, moderate; 5, severe) to describe local adverse effects (ie, pain, rash, itching, swelling) and systemic adverse effects (ie, fatigue, headache, dizziness, nausea, myalgia, fever, need for pain medication).

### Randomization

Patients were randomized 1:1 to receive a third dose of the same, previously administered, mRNA vaccine (mRNA-1273 or BNT162b2) or a single dose of the vector vaccine (Ad26COVS1). Randomization was stratified by maintenance immunosuppressive regimen calcineurin inhibitor (CNI) vs co-stimulation blockade (belatacept). To ensure that comparison groups were approximately the same size, we applied block randomization with a block size of 4 to balance participants randomized to each group.

### Procedures

Participants received a single intramuscular dose of an mRNA-based vaccine (BNT162b2 or mRNA-1273) or a vector-based vaccine (Ad26COVS1) within 28 days of being screened for study participation. A follow-up visit with each participant was conducted on days 29 to 42 after vaccination to evaluate their immune response level and for a structured evaluation of any adverse reactions.

### Laboratory Measurements

#### Antibody Response Against SARS-CoV-2

Antibody response was evaluated using the Elecsys^®^ Anti-SARS-CoV-2 enzyme immunoassay (Roche Diagnostics), which tests for the receptor-binding domain of the SARS-CoV-2 spike protein (cutoff, ≥0.8 U/mL; per manufacturer’s instructions). Samples with levels below the limit of detection were set to 0.2 U/mL. The measured U/mL are highly correlated with the World Health Organization’s International Standard BAU/mL (*r* = 0.9996; U/mL = 0.972 × BAU/mL; per manufacturer’s instructions). The functional neutralization capacity of the antibody response was assessed using the surrogate virus neutralization test (sVNT) cPass^™^ (GenScript). The sVNT was used according to the manufacturer’s instructions (cutoff, ≥30% signal inhibition) for test result positivity indicating the presence of neutralizing antibodies.

### Cellular Immune Response

We used QuantiFERON SARS-CoV-2 assays (Qiagen), a SARS-CoV-2 spike protein-specific interferon-γ release assay (IGRA), to assess T-cell response at screening and 4 weeks after the third vaccination. Assays were processed according to the manufacturer’s instructions. Results less than 0.005 IU/mL were set to 0.005 IU/mL. The sum of the results from the 2 antigen tubes (Ag1, Ag2) is reported throughout the Results section of this article. Signals greater than 0.1 IU/mL were considered to be positive. This cutoff was derived as the mean plus 2 SDs from prepandemic controls (n = 13).

### Torque Teno Virus

Torque teno virus (TTV) DNA was quantitated from 200 μL of plasma by real-time polymerase chain reaction, as previously described. The TTV serves as a surrogate for the overall state of the immune system and viral control in immunosuppressed individuals.^22^

### Statistical Analysis

Demographics of the study participants were summarized as medians (IQRs) for continuous covariates and absolute and relative frequencies for categorical parameters. A Fisher exact test was used to test for statistically significant differences in the rate of responders (ie, antibodies >0.8 U/mL threshold) between groups. Statistically significant difference in antibody and interferon-γ concentration between the 2 treatment arms was assessed using Wilcoxon rank sum tests. Patient-specific change in interferon-γ concentration after the second and the third vaccination was evaluated using a Wilcoxon signed rank test. Univariable logistic regression models were used to test the association of patient characteristics with seroconversion after the third vaccination. All secondary end point analyses were conducted in an exploratory fashion and no adjustment for multiple testing was performed. Based on available literature, there was no preliminary data available to assess effect size of a third vaccination nor heterologous vaccination using a vector dose after primary vaccination with an mRNA vaccine. Fisher exact test was used to test for statistically significant differences in patient reported adverse effects, and Cramér V was calculated as a measure of association. Based on available literature,^17^ we estimated a response rate of 30% for a third vaccination with an mRNA vaccine. Enrollment of 100 patients per group (expecting a drop-out rate of ≤5%) would provide 80% power (alpha error, 5%) to detect a statistically significant increase in the response rate for the vector vaccine of 20% compared with the mRNA vaccines. Statistical tests were 2-tailed and a *P* value < .05 was considered statistically significant. Data analyses were performed from August 17 to August 31, 2021, using R, version 4.1 (The R Foundation for Statistical Computing).

## Results

### Participants

**Figure 1.**
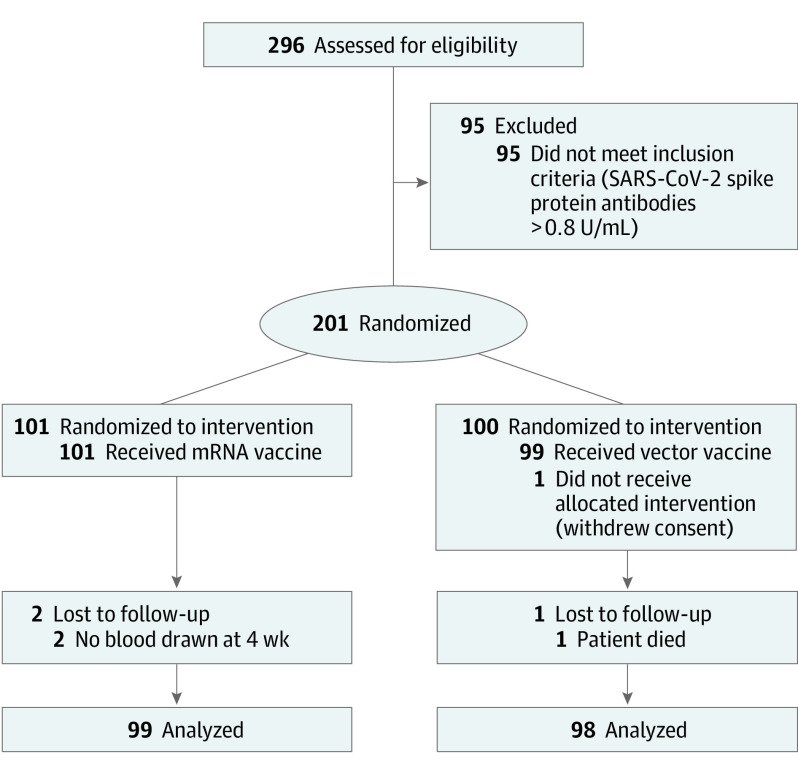
CONSORT Flowchart of Kidney Transplant Recipients Included in Study Cohort

The study population totaled 197 kidney transplant recipients (mean age [SD], 61.2 [12.4] years; 115 [58%] men; 82 [42%] women; race and ethnicity were not recorded) who had not experienced an antibody response to 2 prior mRNA vaccinations. Of the 201 patients that had been enrolled and randomized to receive a third dose of a homologous (mRNA) vaccine (n = 101) or a heterologous (Ad26COVS1) vaccine (n = 100), 2 participants in the mRNA group were lost to follow-up, 1 participant in the vector group withdrew consent before vaccination, and another in the vector group died of a myocardial infarction 4 weeks after vaccination. Therefore, a total of 197 patients were available for the prespecified analyses of the primary end point (Figure 1). Patient characteristics are provided in the Table. There were no statistically significant differences between groups at baseline.

**Table.** Baseline Characteristics of Study Cohort

Variable	Third dose vaccine type, No. (%)^a^	
	mRNA	Vector
Participants, No.	99	98
Mean (SD) age, y	61.2 (13.1)	61.2 (11.8)
Sex		
Female	42 (42)	40 (41)
Male	57 (58)	58 (59)
Time since KTX, y	4.68 (2.41-8.25)	4.51 (1.7-7.29)
No. of KTX		
1	75 (76)	78 (80)
2	17 (17)	15 (15)
3	5 (5)	4 (4)
4	2 (2)	0 (0)
5	0 (0)	1 (1)
Donor type (living)	16 (16)	19 (19)
Initial vaccinations (mRNA-1273)	29 (29)	33 (34)
Maintenance immunosuppression		
Belatacept, MMF, steroids	7 (7)	6 (6)
Belatacept, azathioprine, steroids	0 (0)	1 (1)
Cyclosporin A, MMF steroids	1 (1)	4 (4)
Cyclosporin A, MMF	3 (3)	1 (1)
Cyclosporin A, azathioprine, steroids	1 (1)	0 (0)
MMF, steroids	1 (1)	1 (1)
Tacrolimus		
Leflunomide, steroids	0 (0)	1 (1)
MMF, steroids	77 (78)	74 (76)
MMF	2 (2)	5 (5)
Azathioprine, steroids	4 (4)	3 (3)
Steroids	3 (3)	2 (2)
ATG in past year	1 (1)	2 (2)
Nontriple immunosuppression	9 (9)	9 (9)
TTV copies/mL	3.4×10^5^ (1.1×10^4^-1.7x10^7^)	3.8×10^5^ (5.1×10^4^-1.2×10^7^)
Time between second and third vaccination, d	78 (56-87)	82 (57-90)
Time between third vaccination and follow-up visit, d	29 (28-32)	30 (28-33)

Abbreviations: ATG, antithymocyte globulin; KTX, kidney transplant; MMF, mycophenolate mofetil; TTV, torque teno virus.

^a^ Data are presented by frequency (%) or by median (IQR).

### Primary End Point

Four weeks after the third vaccination, 76 patients had developed antibodies against the SARS-CoV-2 spike protein (>0.8 U/mL), for an overall response rate of 39%. There was no statistically significant difference between the homologous and heterologous vaccination strategies, with a response rate of 35% vs 42% for mRNA vs vector vaccine (OR, 1.31; 95% CI, 0.71-2.44; *P* = .38).

### Secondary End Points

**Figure 2.**
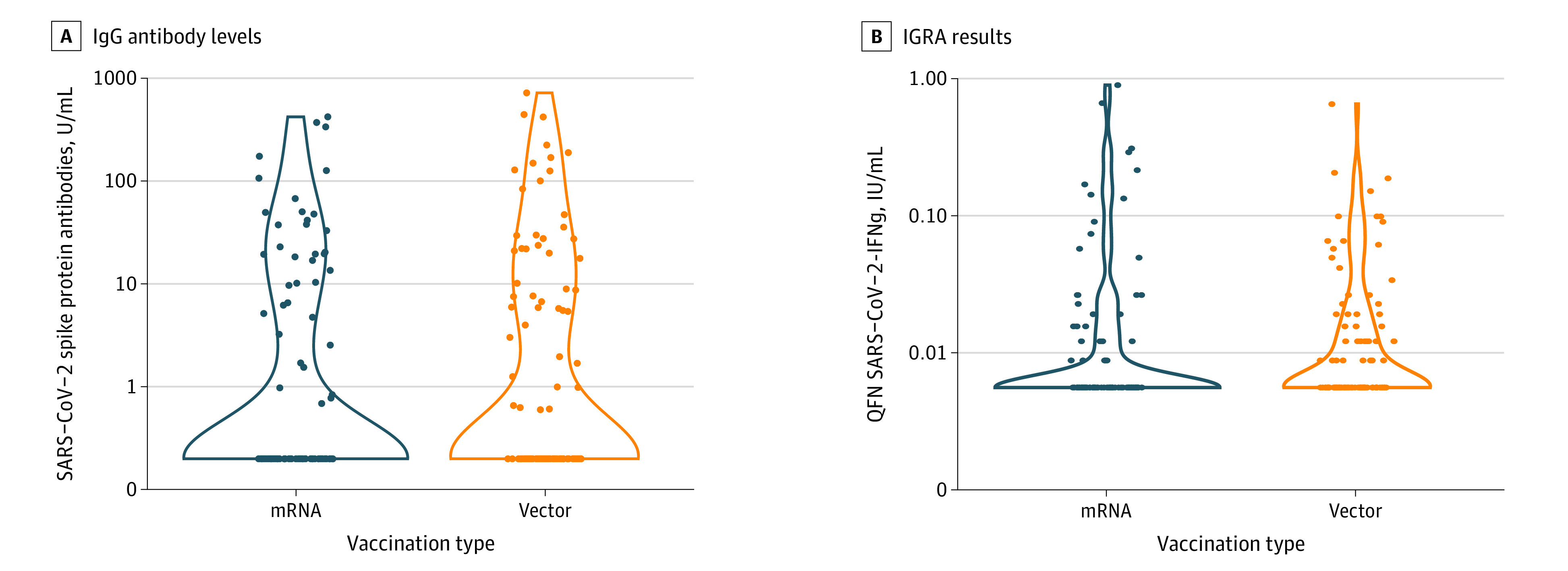
Antibody Levels and Results of Interferon-γ Release Assays (IGRA), 4 Weeks After Third SARS-CoV-2 Vaccination, by Vaccine Type A, The IgG antibody levels of participants in the mRNA vs vector vaccine groups. B, The IGRA results of participants in the mRNA vs vector vaccine groups. IFNg refers to interferon-γ (gamma); IgG, immunoglobulin G; and QFN, the QuantiFERON SARS-CoV-2 assay (Qiagen).

Applying the antibody cutoff levels ( >15 U/mL; >100 U/mL; >141 BAU/mL; and >264 BAU/mL), the overall response rate at 4 weeks was 22%, 8%, 6%, and 3%, respectively, with no statistically significant differences between groups (15 U/mL, 21% vs 23% [OR, 1.14; 95% CI, 0.55-2.36; *P* = .73]; 100 U/mL, 6% vs 10% [OR, 1.76; 95% CI, 0.55-6.14; *P* = .31]; 141 BAU/mL, 4% vs 7% [OR, 1.82; 95% CI, 0.45-8.78; *P* = .37]; 264 BAU/mL, 3% vs 3% [OR, 1.01; 95% CI, 0.13-7.74; *P* > .99] for mRNA and vector vaccine, respectively). Figure 2 shows antibody levels following the third vaccination with mRNA and vector vaccines; mean antibody levels for mRNA and vector vaccine were 22 and 33 U/mL, respectively. Only 22% of the positive antibody responses (>0.8 U/mL) showed functional neutralizing capacity (eFigure 1 in the Supplement 2). There was no statistically significant difference between the mRNA and vector vaccine groups regarding antibodies with neutralizing capacity (6% vs 11% for mRNA and vector vaccine, respectively; OR, 1.95; 95% CI, 0.63-6.72; *P* = .22). eFigure 1 in the Supplement 2 shows the correlation of immunoassay and neutralizing capacity tests.

The overall T-cell response assessed by IGRA was low with only 17 KTRs showing a positive reactivity against the SARS-CoV-2 spike protein (9 in the mRNA and 8 in the vector group; OR, 0.86; 95% CI, 0.27-2.64; *P* = .80). There was no statistically significant difference in reactivity between groups after the third vaccination (mean, 0.049 IU/mL and 0.037 IU/mL for mRNA and vector groups, respectively; *P* = .19; Figure 2). However, we observed an increase in reactivity between the screening and follow-up time points (mean, 0.028 IU/mL and 0.043 IU/mL, respectively; *P* = .02; eFigure 2 in the Supplement 2).

### Predictors Associated With Vaccination Response

**Figure 3.**
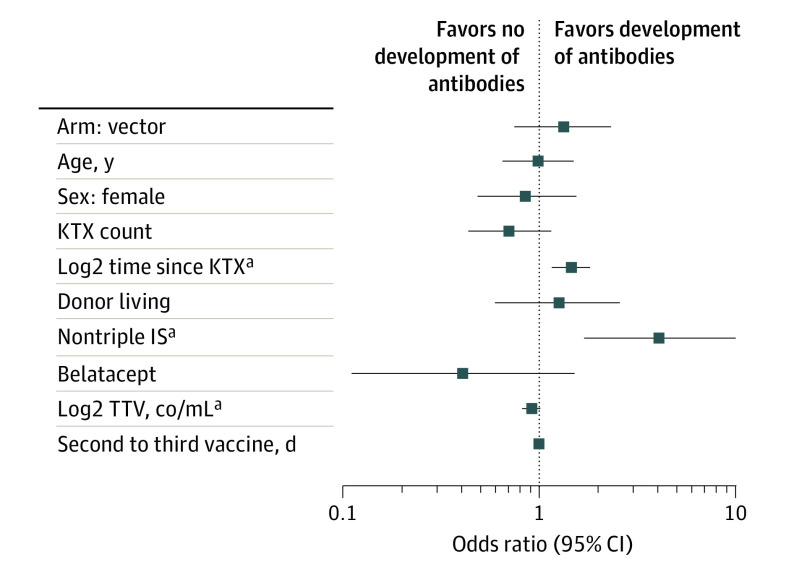
Forest Plot of Predictors for Vaccine Response After the Third Dose of SARS-CoV-2 vaccines ^a^Time since KTX, nontriple IS, and TTV levels showed a significant association with response to third dose. Co denotes copies; IS, immunosuppression; KTX denotes kidney transplant; and TTV, torque teno virus.

Figure 3 provides an overview of predictors associated with response to the third vaccination. Type of vaccine received as the third dose was not associated with antibody development after vaccination (OR, 1.32; 95% CI, 0.74-2.35; *P* = .35). Patients not on triple-maintenance immunosuppression had a significantly higher chance of developing antibodies compared with patients on triple immunosuppression (OR, 3.59; 95% CI, 1.33-10.75; *P* = .01). Most patients (92%) were on a CNI-based immunosuppressive regimen; only (7%) received belatacept-based immunosuppression. Only 21% of patients on belatacept developed SARS-CoV-2 spike protein antibodies; however, there was no statistically significant difference in the response rate of patients on belatacept (OR, 0.41; 95% CI, 0.09-1.37; *P* = .18). In line, lower TTV levels were associated with response to the vaccine (OR, 0.92; 95% CI, 0.88-0.96; *P* < .001 per doubling of TTV copies [co] /mL). Furthermore, longer time since last kidney transplant was also associated with a higher chance of developing an antibody response after the third dose (OR, 1.44; 95% CI, 1.15-1.83; *P* = .002 per doubling of years). Recipient age, sex, donor type, number of transplants, and time between second and third vaccinations did not show a statistically significant association.

### Adverse Events and Reactogenicity

Five serious adverse events occurred in the 4-week follow-up period after vaccination: 1 patient died of a myocardial infarction (vector group); 2 patients were hospitalized for urinary tract infections (1 in each group); 1 patient developed thrombophlebitis 3 weeks after vaccination (vector group) and required systemic anticoagulation (laboratory work-up results showed no signs of thrombocytopenia and platelet factor 4 antibodies were negative)^23^; and 1 patient was hospitalized for treatment of symptomatic hypokalemia (mRNA group).

The third vaccination was tolerated well, with 1% to 2% of patients reporting severe fatigue, fever, myalgia, or pain. Reactogenicity was widely similar between vector and mRNA groups; however, more patients in the mRNA vaccination group reported pain at the injection site (Cramér V, 0.22; 95% CI, 0.12-0.36; *P* = .01; eFigure 3 in the Supplement 2).

## Discussion

The study findings show that a third dose of vaccine induced a SARS-CoV-2 antibody response in 39% of KTRs who had no seroconversion after 2 doses of mRNA vaccines. The observed difference in the antibody response rate at 4 weeks after the third dose—35% vs 42% for mRNA and vector, respectively—was not statistically significant. Quantitative analysis of antibody levels, as well as evaluation of the cellular immune response, also showed no statistically significant difference between groups.

The antibody levels found in KTRs who developed an immune response were considerably lower compared with levels observed in the general population.^14^ When we applied the higher cutoff levels used in the literature^19,20,21^ —shown to be highly correlated with neutralizing antibody as a surrogate for protective immunity—the success rate of the third dose was much lower. These patients with low-level antibody responses may remain at risk for SARS-CoV-2 infection. Accordingly, only 22% of the participants demonstrating seroconversion had antibodies with neutralizing capacity; none had a positive test result for SARS-CoV-2 nucleocapsid antibodies at 4 weeks after the third dose. Importantly, 1 participant with a low-level antibody response (5.91 U/mL) and 1 participant without any antibody response later died of COVID-19. Longer follow-up of this study’s participants may reveal differences in the trajectories of antibody levels among those who received the mRNA vs the vector vaccine, and low-level antibody responses may require additional dosing (ie, a fourth vaccination) to achieve protective immunity.^19^

After the third vaccination, SARS-CoV-2 specific T-cell response assessed by IGRA was low overall, with no difference between groups. A reduced T-cell response following SARS-CoV-2 vaccination has previously been described and further reflects efficacy of pharmacologic T-cell suppression following kidney transplant.^24^ Results of IGRA showed a positive response to 2 doses of SARS-CoV-2 vaccine among individuals with immunocompetence (eFigure 4 in the Supplement 2) but sensitivity may be reduced in individuals who are immunosuppressed, which may explain the lower response rates compared with results of enzyme-linked immunospot assays.^9^ However, the clinical relevance of low-level T-cell responses remains unclear.

Most patients in this trial were taking a CNI-based triple-immunosuppressive regimen—previously identified as a risk factor for vaccine nonresponse after 2 doses of mRNA vaccine.^5,9^ A T-cell−focused immunosuppressive regimen effectively inhibits a de novo immune response against the transplanted organ; however, it also impairs viral control in the absence of a memory immune response that is dependent on immunization prior to the establishment of immunosuppression.^25,26^

Patients who are in the early period after transplant are at higher risk of not developing protective immunity after 2 doses of mRNA vaccine.^5,9^ This higher risk may be primarily attributable to higher levels of immunosuppression early after transplant. Accordingly, TTV levels, which mainly reflect T-cell−mediated viral control and serve as surrogate for the overall level of immunosuppression, were associated with antibody response to a third vaccination.^27^ An apathogenic virus, TTV is increasingly being recognized as marker for the overall state of the immune system following organ transplant. Higher TTV levels have been identified as a risk factor for infection after transplant.^22^

Overall, a third dose of both vaccines was well tolerated. Reported adverse events were not related to vaccination but rather, reflect the overall disease burden among KTR. More participants in the mRNA vaccine group reported local pain at the injection site. Higher reactogenicity of mRNA vaccines as a booster dose after primary vaccination with a vector vaccine in heterologous vaccination has been previously described.^28^ In the present study, systemic symptoms after vaccination were comparable between the mRNA and vector vaccine groups.

### Limitations

Limitations of the study include the sample size of 200, which would have allowed differences in response of more than 20% to be detected. On the other hand, the core strength of the study was its randomized clinical trial design, comparing homologous and heterologous vaccination. The inclusion of only KTRs without a detectable immune response after 2 doses of mRNA vaccine selected a high-risk population with severely impaired immune response to vaccination. This inclusion criteria allowed for a comparison in the immune response of homologous vs heterologous vaccination by measuring antibody levels in a small cohort as surrogate marker for immunization, rather than infection and clinical outcomes in a much larger trial of the general population. However, there is currently insufficient data on the association of antibody levels (including neutralizing capacity) with clinical outcomes (ie, protection from infection and severe disease). Thus, serologic tests can only serve as a surrogate for protective immunity, and real-world data suggest an almost 80% reduction in the incidence of symptomatic COVID-19 among vaccinated solid organ transplant recipients.^29^ External validity of the study is limited to the study population, which was predominantly of White race.

## Conclusions

This randomized clinical trial found that more than 1 in 3 KTRs without an immune response against SARS-CoV-2 after 2 doses of an mRNA vaccine developed antibodies against the spike protein after the third dose. However, only 22% of those showing seroconversion had antibodies with neutralizing capacity. The heterologous vaccination strategy was safe and well tolerated but did not result in a statistically significant difference in the humoral immune response to a third dose of SARS-CoV-2 vaccine after 4 weeks.

Strategies to improve the immune response in solid organ transplant recipients are urgently needed. Further research is required to determine if additional doses (ie, fourth dose or more) increase the proportion of KTRs developing protective immunity, as observed among initial nonresponders to the hepatitis B vaccine.^30^ Other immunization strategies may include temporarily reducing maintenance immunosuppression or prophylaxis with long-acting recombinant neutralizing antibodies that successfully reduce viral load in patients without protective immunity and which may convey passive immunity for several months.^31^

## Notes

Supplement 1.
Study Protocol.

Supplement 2. **eFigure 1. **Neutralizing capacity of SARS-CoV-2 spike protein antibodies **eFigure 2.** SARS-CoV-2 spike protein specific interferon-gamma release assay results before and four weeks after the third vaccination **eFigure 3.** Reactogenicity following third vaccination **eFigure 4.** Interferon-gamma release assay results in healthy subjects after two doses of mRNA vaccine

Supplement 3. Data Sharing Statement.
