# Supplementary material for: Comparison of SARS-CoV-2 Antibody Response 4 Weeks After Homologous vs Heterologous Third Vaccine Dose in Kidney Transplant Recipients: A Randomized Clinical Trial
Source: JAMA Intern Med. 2021 Dec 20;182(2):1–8. doi: 10.1001/jamainternmed.2021.7372 (PMC8689434; doi:10.1001/jamainternmed.2021.7372)
Supplement: Supplement 1. — Study Protocol. [file jamainternmed-e217372-s001.pdf]

Study Protocol

Version 1.1

06-09-2021

Single blinded randomized controlled trial on BNT162b2 or mRNA-1273 (mRNA) vs Ad26COVS1 (viral vector) in kidney transplant recipients without SARS-CoV-2 spike protein antibodies following full vaccination against COVID-19 (BOOST-TX)

EudraCT: 2021-002927-39

Principal investigator: Roman Reindl-Schwaighofer, MD, PhD

Sub investigator: Andreas Heinzel, MSc

Sponsor: Medical University of Vienna (Sponsor representative: Rainer Oberbauer, MD, PhD)

Division of Nephrology and Dialysis

Department of Internal Medicine III

Medical University of Vienna

1. List of abbreviations

COVID-19… Corona Virus Disease 2019

CNI… Calcineurin inhibitor

CRF… Case Report Form

EMA… European Medicines Agency

GCP… Good Clinical Practice

i.m. … intramuscular

mRNA… messenger Ribonucleic Acid

SAE… Serious Adverse Event

SARS-CoV-2… Severe Acute Respiratory Syndrome Coronavirus type 2

SUSAR… Suspected Unexpected Serious Adverse Reaction

2. Table of Contents

1. *List of abbreviations* 2

2. *Table of Contents* 3

3. *Background* 6

Study rationale 7

4. *Study objective:* 7

Primary objective (Hypotehsis) 7

Primary Endpoint 7

Secondary endpoints 8

5. *Study design* 8

Study population 9

Inclusion criteria 9

Exclusion criteria 9

6. *Methodology* 10

Subject enrollment: 10

Patient screening 10

Randomization 11

Blinding 11

Treatment 12

Study medication, Dosage and Administration 12

Treatment arm 1 (“more of the same”): 12

Treatment arm 2 (“mix and match”): 13

Study-drug delivery and drug storage conditions 13

Follow-up: 13

Study duration 14

End of study 14

Withdrawal of subject 14

Additional study related procedures 14

Laboratory testing 15

Clinical data 15

Benefit and risk assessment 15

7. *Adverse event reporting* 16

Definitions 16

Adverse Events 16

Serious Adverse Events 17

Serious Adverse Event reporting procedures 18

Summary of known and potential risks of the study drug 19

8. Statistical analysis *22*
Sample size calculation **22**
Endpoint analysis **23**
9. Documentation and data management *25*
Documentation and data management **25**
Case report form (CRF) 25
Safekeeping **26**
Quality control and quality assurance **26**
Monitoring 26
Inspections 27
Reporting and publication **27**
10. Ethical and Legal aspects *27*
Informed consent of subjects **27**
Acknowledgement / approval of the study **28**
Insurance **29**
Ethics and good clinical practice (GCP) **29**
11. References *30*

Periods	Name	Screening	Treatment	Follow-up				
Visits	Number	1	2	3	4	5	6	7
	Name	Screening	Treatment	Follow up 1	Follow up 2	Follow up 3	Follow up 4	Follow up 5
	Time	within 28 days prior to vaccination	Day 1	Day 29-42 (4 weeks after treatment)	Day 57-70 (8 weeks after treatment)	Day 85-98 (12 weeks after treatment)	Day 168-196 (24 weeks after treatment)	Day 336-364 (48 weeks after treatment)
Informed consent		x						
Inclusion / Exclusion Criteria		x						
Medical History		x						
Concomitant medication		x						
SARS-CoV-2 Antibodies		x		x	x	x	x	x
T-cell response		x		x				
Pregnancy test		x	x					
Adverse events			x	x	x	x	x	x

Table 1: Visit and Assessment scheduled

3. Background

Kidney transplant recipients are at high risk for a severe COVID-19 disease course and therefore prioritized to receive early vaccination against SARS-CoV-2 (1, 2). In general, the immune response to vaccination is reduced in immunosuppressed individuals including kidney transplant recipients. A recent study from Israel confirmed that only 37.5% of kidney transplant recipients developed detectable antibodies against the spike protein following full vaccination with BNT162b2 (Pfizer-BioNTech) (3). The type of maintenance immunosuppression may have a significant impact on the immune response in kidney transplant recipients. Patients treated with co-stimulation blockade (Belatacept) as maintenance immunosuppression showed even lower immunization rates of only 5.7 % (4).

Several strategies have been proposed to improve response to SARS-CoV-2 vaccination including additional dosing and mixing different vaccine types to broaden the presented antigen repertoire (heterologous vaccination or “mix-and-match strategy”) (5). Recent data show a higher reactogenicity of heterologous boosting in the general population (6). In the European Union there are currently two different classes of SARS-CoV-2 vaccines available: a) mRNA (mRNA-1273 [Moderna Biotech] and BNT162b2 [Pfizer-BioNTech]) and b) viral vector-based vaccines (ChAdOx1 [AstraZeneca] and Ad26COVS1 [Jansen]). Of these Ad26COVS1 is the only vaccine that has been shown to induce a protective immune response after a single dose (7). Kidney transplant recipients in Austria have primarily been vaccinated with mRNA-based vaccines.

Study rationale

More than half of all kidney transplant recipients do not develop SARS-CoV-2 antibodies following full vaccination with a mRNA-based SARS-CoV-2 vaccine. However, there is currently no data available to guide immunization strategies in these patients. A “mix and match” strategy combining different types of vaccines has been proposed to improve immune response following vaccination, but efficacy of such a strategy in kidney transplant recipients has not yet been evaluated.

4. Study objective:

The primary objective of this study is to test if a “mix and match” strategy (using a viral vector-based vaccine) results in a better antibody response against the SARS-CoV-2 spike protein compared to an additional dose of mRNA vaccine (“more-of-the-same”) in patients who did not develop antibodies following full vaccination with a mRNA vaccine.

Primary objective (Hypotehsis)

We specifically hypothesize that a single dose of the viral vector-based vaccine Ad26COVS1 [Jansen] results in a higher rate of antibody response compared to a third dose of the previously used mRNA vaccine (mRNA-1273 [Moderna Biotech] or BNT162b2 [Pfizer-BioNTech]).

Primary Endpoint

- Number of patients presenting a positive humoral immune response (antibody), assessed with the Roche ELISA test using the manufacturer recommended cutoff of

0.8U/ml, to theSARS-CoV-2 vaccine at 4 weeks after the vaccination. The decision to perform a responder analysis is motivated by the fact that currently the exact relationship between anti-SARS-CoV-2 antibody concentration and protective immunity is not known.

Secondary endpoints

- Number of patients presenting a positive humoral immune response (antibody), assessed with the Roche ELISA test using the manufacturer recommended cutoff of 0.8U/ml, to the SARS-CoV-2 vaccine at 8, 12, 24, 48 weeks after the vaccination.
- Number of detectable antibodies (U/ml measured by the Roche ELISA test) at 4, 8, 12,24 and 48 weeks after vaccination.
- Number of patients presenting a positive cellular immune response defined as doubling of extracellular IFNg concentration in the plasma after ex-vivo re-stimulation with a SARS-CoV-2 spike protein peptide pool at 4 weeks after the vaccination.
- Strength of the cellular response to SARS-CoV-2 spike protein peptides measured in terms of the extracellular IFNg concentration after ex-vivo re-stimulation with a peptide pool at 4 weeks after the vaccination.
- incidence of COVID-19 disease within 24 and 48 months after vaccination

5. Study design

This is a single center, single blinded, randomized study. Only the patient will be blinded for the type of vaccine administered. Based on sample-size calculations (see below) a total of 200 patients will be enrolled into the study.

Study population

The study population comprises kidney transplant recipients without detectable SARS-CoV-2 spike protein antibodies at least 4 weeks after the second dose of a mRNA based vaccine.

Inclusion criteria

- patient has received a kidney transplantation
- full SARS-CoV-2 vaccination with mRNA vaccine (two doses) at least 4 weeks before screening
- > 18 years of age
- no SARS-CoV-2 spike protein antibodies at least 4 weeks after the second dose of an mRNA vaccine

Exclusion criteria

- acute illness with fever
- Prior documented infection with SARS-CoV-2
- triple anticoagulation therapy
- Subject is currently enrolled in or has not yet completed at least 30 days since ending other investigational device or drug trial(s), or subject is receiving other investigational agent(s)
- Subject has known sensitivity or intolerance to any of the products to be administered for the purpose of this study

- Subject has any kind of disorder that compromises the ability of the subject to give written informed consent and/or to comply with the study procedures
- Subject is pregnant or breast feeding

Females of childbearing potential may participate in this clinical trial, although a negative pregnancy test is required during screening.

6. Methodology

Subject enrollment:

All subjects must personally sign and date the consent form before any screening procedures are performed. Patients are eligible for enrollment when they have satisfied all of the inclusion/exclusion criteria. Following enrollment patients will be randomized to one of the treatment groups. All participants who enter into the screening period for the study (after signing the informed consent) will receive a personal identification number. This number will be used to identify the subject throughout the trial and must be used on all study documentation related to the subject. The subject identification number must remain constant throughout the entire trial.

Patient screening

Patients followed at the transplant clinic of the Medical University of Vienna who received two doses of mRNA-based SARS-CoV-2 vaccine will be invited to the study. In the screening phase kidney transplant recipients that are followed at the kidney transplant clinic of the Medical University of Vienna that fulfill the mentioned inclusion criteria will be identified.

Following informed consent, patients will be tested for SARS-CoV-2 spike protein and nucleocapsid antibodies. Screening has to be performed within 4 weeks prior to vaccination.

Randomization

Randomization will be performed as soon as the SARS-CoV-2 antibody status is available.

- Patients included in the study will be randomized 1:1 to receive a third dose of the previously administered mRNA vaccine (mRNA-1273 or BNT162b2) or a single dose of Ad26COVS1
- Randomization will be stratified by the maintenance immunosuppressive regimen calcineurin inhibitor (CNI) vs co-stimulation blockade (Beletacept)
- To ensure that comparison groups will be of approximately the same size a block randomization will be used in order to balance subjects randomized to each group (https://www.meduniwien.ac.at/randomizer/).

Blinding

Patients will be blinded for the treatment.

Prohibited medication

The exclusion criteria describe triple anticoagulation as prohibited medication in this trial.

There are no prohibited therapies during the Post-Treatment Follow-up phase

Treatment

Patients will receive a single dose of the study drug intra muscular (i.m.) within 28 days after screening. Patients will receive a time slot for vaccination following randomization. Vaccination will be performed at the outpatient clinic of the Division of Nephrology and Dialysis at the Medical University of Vienna (chronic hemodialysis unit and pre-transplant clinic).

Study medication, Dosage and Administration

Study medication will be provided by the central pharmacy of the Medical University of Vienna.

Treatment arm 1 (“more of the same”):

active substance: mRNA-1273

trade name: COVID-19 Vaccine Moderna

Manufacturer: ROVI PHARMA INDUSTRIAL SERVICES S.A.

Route of administration: i.m.

Duration: once

Dose: 0.5 ml

or

active substance: BNT162b2

trade name: COMIRNATY COV-19 VAC

Manufacturer: BIONTECH MANUFACTURING GMBH

Route of administration: i.m.

Duration: once

Dose: 0.3 ml

Treatment arm 2 (“mix and match”):

active substance: Ad26COVS1

trade name: COVID-19 Vaccine Janssen

Manufacturer: JANSSEN BIOLOGICS B.V.

Route of administration: i.m.

Duration: once

Dose: 0.5 ml

Study-drug delivery and drug storage conditions

The vaccines are prepared and dispensed by the central pharmacy of the General Hospital of Vienna. All trial substances will be handled and administered according to manufacturer’s recommendations.

Follow-up:

Follow up visits will be scheduled 4, 8, 12,24 and 48 weeks after vaccination at the kidney transplant outpatient clinic to assess the immune response to the vaccination (blood draw for antibody and T-cell measurement). Independent from study visits, patients are routinely followed at the transplant clinic of the Nephrology Department of the Medical University of Vienna at regular intervals including blood draws (creatinine, immunosuppression levels).

Study duration

The response to the vaccination will be evaluated at day 29-42 (visit 3, primary endpoint) as well as day 57-70, day 85-98, day 168-198 and day 336-364 (visit 4, 5, 6 and 7, secondary endpoints).

End of study

The end of the trial is the last visit of the last patient.

Withdrawal of subject

Subjects may prematurely discontinue from the study at any time. Subjects must be withdrawn under the following circumstances:

- at their own request
- if the Investigator feels it would not be in the best interest of the subject to continue
- if the subject violates conditions laid out in the consent form / information sheet or

disregards instructions by the study personal

In all cases, the reason why subjects are withdrawn must be recorded in detail in the CRF Should the study be discontinued prematurely, all study materials (completed, partially completed and empty CRFs) will be retained.

Additional study related procedures

Table 1 shows summarizes Study visits and Assessments.

Laboratory testing

- SARS-CoV-2 spike protein and nucleocapsid antibodies at Visit 1, Visit 3, Visit 4, Visit 5, Visit 6 and Visit 7 (9ml blood)
- SARS-CoV-2 specific T- cell response at Visit 1 and Visit 3 (3x8 ml blood)

Antibodies will be measured by the Department of Laboratory Medicine using clinically validated ELISA tests (Roche, Switzerland). SARS-CoV-2 specific T-cell responses will be assessed by QuantiFERON assays with SARS-CoV-2 specific peptides at the research laboratory of the Division of Nephrology (Quiagen, Netherlands).

Clinical data

- Baseline clinical data will be recorded at visit 1 (current medication including maintenance immunosuppression, medical history including date of transplantation) using the CRF document.
- Adverse events will be assessed at visits 2, 3, 4 and 5

Benefit and risk assessment

Patients with immunosuppression are considered to be at great risk for a severe disease course of COVID-19 an additional vaccination will increase their chance to develop a protective immune response. The risk for adverse events following a third vaccination is low compared to the risk associated with a SARS-CoV-2 infection in this population. All study drugs have been approved by the European Medicines Agency (EMA).

7. Adverse event reporting

Definitions

Adverse Events

An adverse event is by definition “any untoward medical occurrence in a patient or clinical investigation subject administered a pharmaceutical product and that does not necessarily have a causal relationship with this treatment.” (International Conference on Harmonisation [ICH] Guideline for Good Clinical Practice).

- Worsening of a pre-existing medical condition (e.g., cancer, diabetes, migraine headaches, gout) should be considered an adverse event if there is either an increase in severity, frequency, or duration of the condition or an association with significantly worse outcomes.
- Interventions for pretreatment conditions (e.g., elective surgery) or medical procedures that were planned before study enrollment are not considered adverse events.

The investigator is responsible for reviewing laboratory test results and determining whether an abnormal value in an individual study subject represents a change from values before the study. In general, abnormal laboratory findings without clinical significance (based on the investigator's judgment) should not be recorded as adverse events. However, laboratory value changes requiring therapy or adjustment in prior therapy are considered adverse events.

Serious Adverse Events

A serious adverse event (SAE) is defined as an adverse event that:

- is fatal
- is life threatening (places the subject at immediate risk of death)
- requires in-patient hospitalization or prolongation of existing hospitalization
- results in persistent or significant disability/incapacity
- is a congenital anomaly/birth defect
- other significant medical hazard

A hospitalization meeting the regulatory definition for “serious” is any inpatient hospital admission that includes a minimum of an overnight stay in a health care facility. Any adverse event that does not meet one of the definitions of serious (e.g., emergency room visit, outpatient surgery, or requires urgent investigation) may be considered by the investigator to meet the “other significant medical hazard” criterion for classification as a serious adverse event. Examples include allergic bronchospasm, convulsions, and blood dyscrasias.

Reporting procedures for all Adverse Events

The investigator is responsible for ensuring that all adverse events (as defined above) observed by the investigator or reported by subjects are properly captured in the subjects’ medical records.

In addition, the investigator is responsible for ensuring that, for those subjects randomized into the study, all adverse events captured on the subjects’ medical records (as specified

above) are reported on the case report form. This collection period will be from the time of administration of the study drug until the end of the study.

The following adverse event attributes must be assigned by the investigator:

- adverse event diagnosis or syndrome(s) (if known, signs or symptoms if not known)
- event description (with detail appropriate to the event)
- dates of onset and resolution
- severity
- assessment of relatedness to investigational product
- action taken

The relationship of the adverse event to the investigational product will be assessed by means of the question: “Is there a reasonable possibility that the event may have been caused by the investigational product?” The investigator should respond to this question with either Yes or No.

Medically significant adverse events considered related to the investigational product by the investigator will be followed until resolved or considered stable.

Adverse events will be reported in the publication arising from this study.

Serious Adverse Event reporting procedures

Serious adverse events will be collected and recorded from the date the informed consent is signed through the end of the study. If a serious adverse event occurs before administration

of the investigation product, the relationship of the adverse event to study screening is to be assessed. If a subject is permanently withdrawn from treatment because of a serious adverse event, this information must be included in the initial or follow-up Serious Adverse Event Report Form as well as the Case Report Forms.

A SAE that is a known and previously described side effect of the treatment will be reported to the Austrian Federal Office for Safety in Health Care as well as the responsible ethics committee once a year in the form of a Development Safety Update Report (DSUR). Events that are suspected unexpected serious adverse reactions (SUSARs) will be reported to the responsible ethics committee and the European Medicines Agency. Fatal SUSARs will be reported as soon as possible, but latest within 7 days and non-fatal SUSARs within 15 days.

Summary of known and potential risks of the study drug

All three study medications are approved by the EMA for vaccination against SARS-CoV-2 in individuals over 16 years of age. Overall safety profiles are excellent with over a billion doses administered worldwide as of May 19,2021 (ourworldindata.org). An overview of undesirable effects and respective frequencies for each study drug are reported below:

COVID-19 Vaccine Moderna [mRNA-1273, Moderna]:

System organ class	undesirable effect	Frequency
Blood and lymphatic system disorders	Lymphadenopathy	very common
Immune system disorders	Anaphylaxis, Hypersensitivity	unknown

Nervous system disorders	Cephalea	very common
	Acute Peripheral facial palsy	rare
Gastrointestinal disorders	Nausea/Emesis	very common
Skin and subcutaneous tissue disorders	Rash	common
Musculoskeletal and connective tissue disorders	Myalgia, Arthralgia	very common
General disorders and administration site conditions	Pain at Injection site, Tiredness, Chills, Fever, Swelling at injection site	very common
	Erythema at Injection site, Urticaria at injection site, Rash at injection site	common
	Itching at injection site	uncommon
	Facial swelling	rare

Comirnaty [BNT162b2, PfizerBioNTech]

System organ class	undesirable effect	Frequency
Blood and lymphatic system disorders	Lymphadenopathy	uncommon
Immune system disorders	Anaphylaxis, Hypersensitivity	unknown
Psychiatric disorders	Sleeplessness	uncommon

Nervous system disorders	Cephalea	very common
Gastrointestinal disorders	Nausea	common
Skin and subcutaneous tissue disorders	Arthralgia, Myalgia	very common
	Pain in the extremities	uncommon
General disorders and administration site conditions	Paint at the injection site, tiredness, chills, fever, swelling at the injection site	very common
	Redness at injection site	common
	Malaise, itching at injection site	uncommon

COVID-19 Vaccine Janssen [Ad26.COV2-S, Johnson&Johnson]

System organ class	undesirable effect	Frequency
Immune system disorders	Hypersensitivity, Urticaria	rare
Nervous system disorders	Anaphylaxis	not known
	Headache	very common
	Tremor	uncommon
Respiratory, thoracic and mediastinal	Cough	common
	Sneezing, oropharyngeal pain	uncommon
Gastrointestinal disorders	Nausea	common
Skin and subcutaneous tissue disorders	Rash	uncommon
	Myalgia	very common

Musculoskeletal and connective tissue disorders	Arthralgia	common
	Muscular weakness, back pain, limb pain	uncommon
General disorders and administration site conditions	Tiredness, pain at injection site	very common
	Fever, erythema at injection site, swelling at injection site, chills	common
	Asthenia, malaise	uncommon

Very common: ≥1/10

Common: ≥1/100 to <1/10

Uncommon: ≥1/1,000 to <1/100

Rare: ≥1/10,000 to <1/1,000

Very rare: <1/10,000

Not known: cannot be estimated from the available data

8. Statistical analysis

Sample size calculation

For testing the primary study endpoint (positive antibody to the SARS-CoV-2 vaccine) we assume a response rate of 30% and 50% in the mRNA and the viral vector-based vaccine treatment arms and expect a dropout rate of a maximum of 5%. Currently, no data on a third vaccination in kidney transplant recipients exist. The 30% response rate for the homologous vaccination group has been chosen expecting a slightly lower response rate to the third vaccination in kidney transplant recipients who did not respond to the standard 2-dose vaccination regime compared to the initial response rate of kidney transplant recipients to

the standard 2-dose vaccination regime (37% response rate). The 50% response rate estimate for the heterologous vaccination groups is based on early data from studies investigating heterologous vaccination which showed a higher reactogenicity after the heterologous vaccination. Enrollment of 100 patients per group will provide 80% power (alpha-error: 5%) to detect a difference in response between the two treatment groups.

Endpoint analysis

For all endpoints a per protocol analysis will be carried out.

For the primary endpoint (positive humoral immune response to SARS-CoV-2 vaccination at 4 weeks after the vaccination) SARS-CoV-2 S IgG antibody test results will be recorded and counts and percentages of patients exceeding the manufacturer defined cutoff of 0.8U/ml in each treatment arm will be reported. A chi-squared test will be employed to test for statistically significant differences in the number of responders (antibody concentration exceeds the manufacturer defined cutoff of 0.8U/ml) between the treatment arms.

Secondary endpoint analysis

All secondary endpoint analysis will be conducted in an exploratory fashion and no adjustment for multiple testing will be performed.

- Number of patients presenting a positive humoral immune response at 8, 12, 24 and 48 weeks after vaccination will be analyzed in the same fashion as the primary endpoint.
- For the analysis of number of detectable antibodies at 4, 8, 12, 24 and 48 weeks after vaccination ELISA readouts (U/ml) will be recorded. At each time point their median and interquartile range in each treatment arm will be determined and a t-test will be

performed to determine if there is a statistically significant difference between the means of the logarithmized ELISA readouts (U/ml) of the two treatment arms.

- For the analysis of number of patients presenting a positive cellular immune response to SARS-CoV-2 vaccination at 4 weeks after the vaccination the extracellular IFNg concentration in a negative control sample and after ex-vivo re-stimulation with a SARS-CoV-2 spike protein peptide pool will be recorded for all patients. Doubling of IFNg concentration after re-stimulation will be considered as positive cellular response to the vaccination. Counts and percentages of patients with positive response will be reported for each treatment arm. A chi-squared test will be employed to test for statistically significant differences in the number of responders between the two treatment arms.
- For the analysis of the strength of the cellular immune response at 4 weeks after vaccination the extracellular IFNg concentration in negative control sample and after ex-vivo re-stimulation with a SARS-CoV-2 spike protein peptide pool will be recorded for all patients. IFNg concentration from the patient specific negative control samples will be subtracted from the re-stimulated samples to normalize for patient specific differences in cellular activation. Median and interquartile range of the normalized IFNg concentrations after re-stimulation will be calculated for both treatment arms and a t-test will be performed to determine if there is a statistically significant difference between the two treatment arms with respect to the means of the logarithmized IFNg concentration after re-stimulation.
- For the analysis of SARS-CoV-2 infection after a third vaccination, counts and percentages of individuals with SARTS-CoV-2 infection by weeks 24 and 48 will be determined

Before carrying out any endpoint analysis the number of study participants either on co-stimulation blockade or CNI as well as the number of patients who were initially vaccinated with either mRNA-1273 or BNT162b2 will be determined. In case of sufficient number of study subjects with different immunosuppressants or initially received vaccines the statistically analysis plan will be updated to introduce additional stratification.

9. Documentation and data management

Documentation and data management

A subject screening and identification Log will be completed for all enrolled subjects with the reasons for exclusion.

Case report form (CRF)

Paper-based CRFs will be used for this trial. All forms have to be completed and must be legible. Entry errors have to be corrected according the ICH-GCP Guidelines.

For each subject enrolled a CRF will be completed and signed by the Investigator or a designated sub-Investigator. If a subject withdraws from the study, the reason must be noted on the CRF. Case report forms are to be completed on an ongoing basis. The entries will be checked by trained personnel (Monitor) and any errors or inconsistencies will be checked immediately. The monitor will collect original completed and signed CRFs at the end of the study. A copy of the completed and signed CRFs will remain on site, while the original data are handed out to the sponsor.

Safekeeping

The Investigator will maintain adequate and accurate records to enable the conduct of the study to be fully documented and the study data to be subsequently verified (according to ICH-GCP "essential documents").

The collected data will be inserted as an entry form into a password protected MySQL database, which is an open-source relational database management system. Only members of the research team will have access to the database. The obtained data in the database will de-personalize (pseudonymize). Depersonalization means that data with identifying information is collected, but the identifying information is then severed from the personal health information data in the research database and is stored separately.

Quality control and quality assurance

Monitoring

The designated monitor will contact and visit the Investigator on a regularly basis and will be allowed to have direct access to all source documents needed to verify the entries in the CRFs and other protocol-related documents provided that subject confidentiality is maintained in agreement with local regulations. It will be the monitor's responsibility to inspect the CRFs at regular intervals according to the monitoring plan throughout the study, to verify the adherence to the protocol and the completeness, consistency and accuracy of the data being entered on them.

Monitoring will be performed by:

Assoc. Prof. Priv.-Doz. Dr. Gregor Bond, PhD; Division of Nephrology and Dialysis, Department of Internal Medicine III, Medical University of Vienna; Spitalgasse 23, 1090 Wien;

Inspections

Upon request, the Investigator will make all study-related source data and records available to a qualified quality assurance auditor mandated by the competent authority inspectors. The main purposes of an audit or inspection are to confirm that the rights and welfare of the subjects have been adequately protected, and that all data relevant for assessment of safety and efficacy of the investigational product have appropriately been reported to the sponsor.

Reporting and publication

The findings of this study will be published by the sponsor (Investigators) in a scientific journal and presented at scientific meetings. The manuscript will be circulated to all co-Investigators before submission. Confidentiality of subjects in reports/publications will be guaranteed.

10. Ethical and Legal aspects

Informed consent of subjects

Following comprehensive instruction regarding the nature, significance, impact and risks of this clinical trial, the patient must give written consent to participation in the study. During the instruction the trial participants are to be made aware of the fact that they can withdraw their consent – without giving reasons – at any time without their further medical care being influenced in any way.

In addition to the comprehensive instructions given to the trial participants by the Investigator, the trial participants also receive a written patient information sheet in comprehensible language, explaining the nature and purpose of the study and its progress.

The patients must agree to the possibility of study-related data being passed on to relevant authorities.

The patients must be informed in detail of their obligations in relation to the trial participants insurance in order not to jeopardize insurance cover.

Acknowledgement / approval of the study

The Investigator will submit this protocol and any related document provided to the subject (such as subject information used to obtain informed consent) to an Ethics Committee (EC) or Institutional Review Board (IRB). Approval from the committee must be obtained before starting the study.

The clinical trial shall be performed in full compliance with the legal regulations according to the Drug Law (AMG - Arzneimittelgesetz) of the Republic of Austria.

An application must also be submitted to the Austrian Competent Authorities (Bundesamt für Sicherheit im Gesundheitswesen (BASG) represented by the Agency for Health and Food Safety (AGES Medizinmarktaufsicht) and registered to the ClinicalTrials.gov database using the required forms.

Insurance

An insurance for each patient enrolled in this study will be taken out. Details on the existing patients insurance are given in the patient information sheet. All subjects will be insured at the Zürich Versicherungs-AG, Schwarzenbergplatz 15, 1010 Wien, Tel.: +43 1 501255 1255, policy number 07229622-2.

Ethics and good clinical practice (GCP)

The Investigator will ensure that this study is conducted in full conformance with the principles of the "Declaration of Helsinki" (as amended at the 64th WMA General Assembly, Fortaleza, Brazil, 2013) and with the laws and regulations of the country in which the clinical research is conducted.

The Investigator of the clinical trial shall guarantee that only appropriately trained personnel will be involved in the study. All studies must follow the ICH GCP Guidelines and the regulatory requirements. Therefore, this study follows the EU Directive embedded in the Austrian drug act.

11. References

1. Weinhandl ED, Wetmore JB, Peng Y, Liu J, Gilbertson DT, Johansen KL. Initial Effects of COVID-19 on Patients with ESKD. J Am Soc Nephrol. 2021.

2. Nair V, Jandovitz N, Hirsch JS, Abate M, Satapathy SK, Roth N, et al. An early experience on the effect of solid organ transplant status on hospitalized COVID-19 patients. Am J Transplant. 2020.

3. Grupper A, Rabinowich L, Schwartz D, Schwartz IF, Ben-Yehoyada M, Shashar M, et al. Reduced humoral response to mRNA SARS-CoV-2 BNT162b2 vaccine in kidney transplant recipients without prior exposure to the virus. Am J Transplant. 2021.

4. Chavarot N, Ouedrani A, Marion O, Leruez-Ville M, Villain E, Baaziz M, et al. Poor Anti-SARS-CoV-2 Humoral and T-cell Responses After 2 Injections of mRNA Vaccine in Kidney Transplant Recipients Treated with Belatacept. Transplantation. 2021.

5. Spencer AJ, McKay PF, Belij-Rammerstorfer S, Ulaszewska M, Bissett CD, Hu K, et al. Heterologous vaccination regimens with self-amplifying RNA and adenoviral COVID vaccines induce robust immune responses in mice. Nat Commun. 2021;12(1):2893.

6. Shaw RH, Stuart A, Greenland M, Liu X, Van-Tam JSN, Snape MD, et al. Heterologous prime-boost COVID-19 vaccination: initial reactogenicity data. Lancet. 2021.

7. Sadoff J, Gray G, Vandebosch A, Cardenas V, Shukarev G, Grinsztejn B, et al. Safety and Efficacy of Single-Dose Ad26.COV2.S Vaccine against Covid-19. N Engl J Med. 2021.
