# Supplementary material for: Comparison of SARS-CoV-2 Antibody Response 4 Weeks After Homologous vs Heterologous Third Vaccine Dose in Kidney Transplant Recipients: A Randomized Clinical Trial
Source: JAMA Intern Med. 2021 Dec 20;182(2):1–8. doi: 10.1001/jamainternmed.2021.7372 (PMC8689434; doi:10.1001/jamainternmed.2021.7372)
Supplement: Supplement 2. — eFigure 1. Neutralizing capacity of SARS-CoV-2 spike protein antibodies eFigure 2. SARS-CoV-2 spike protein specific interferon-gamma release assay results before and four weeks after the third vaccination eFigure 3. Reactogenicity following third vaccination eFigure 4. Interferon-gamma release assay results in healthy subjects after two doses of mRNA vaccine [file jamainternmed-e217372-s002.pdf]

Supplemental Online Content

Reindl-Schwaighofer R, Heinzel A, Mayrdorfer M, et al. Comparison of SARS-CoV-2 antibody response 4 weeks after homologous vs heterologous third vaccine dose in kidney transplant recipients: a randomized clinical trial. *JAMA Intern Med*. Published online December 20, 2021. doi:10.1001/jamainternmed.2021.7372

eFigure 1. Neutralizing capacity of SARS-CoV-2 spike protein antibodies
eFigure 2. SARS-CoV-2 spike protein specific interferon-gamma release assay results before and four weeks after the third vaccination
eFigure 3. Reactogenicity following third vaccination
eFigure 4. Interferon-gamma release assay results in healthy subjects after two doses of mRNA vaccine

This supplemental material has been provided by the authors to give readers additional information about their work.

© 2021 Reindl-Schwaighofer R et al. *JAMA Intern Med.*

Supplemental eFigure 1. Neutralizing capacity of SARS-CoV-2 spike protein antibodies. The functional neutralization capacity of the antibody response is plotted on the x-axis (a ≥30% signal inhibition in the surrogate virus neutralization test indicates the presence of neutralizing antibodies and is visualized by a horizontal line). SARS-CoV-2 specific antibody levels assessed by immunoassay are plotted on the y-axis (antibody levels >15 U/ml, >100 U/ml, >141 BAU/ml and >264 BAU/ml are visualized by vertical lines).

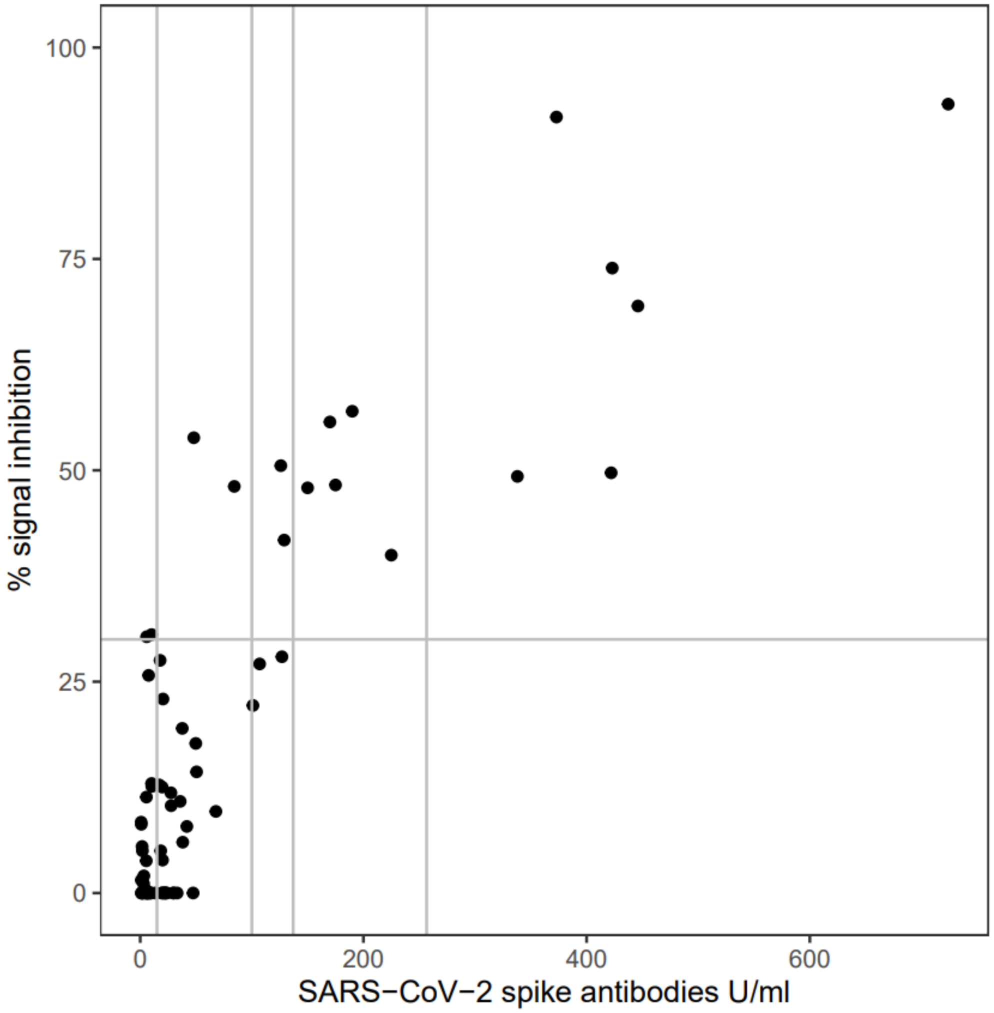

© 2021 Reindl-Schwaighofer R et al. *JAMA Intern Med.*

Supplemental eFigure 2. SARS-CoV-2 spike protein specific interferon-gamma release assay results before and four weeks after the third vaccination. Overall response was low but a statically significant increase of patients interferon-gamma levels after the third vaccination was observed.

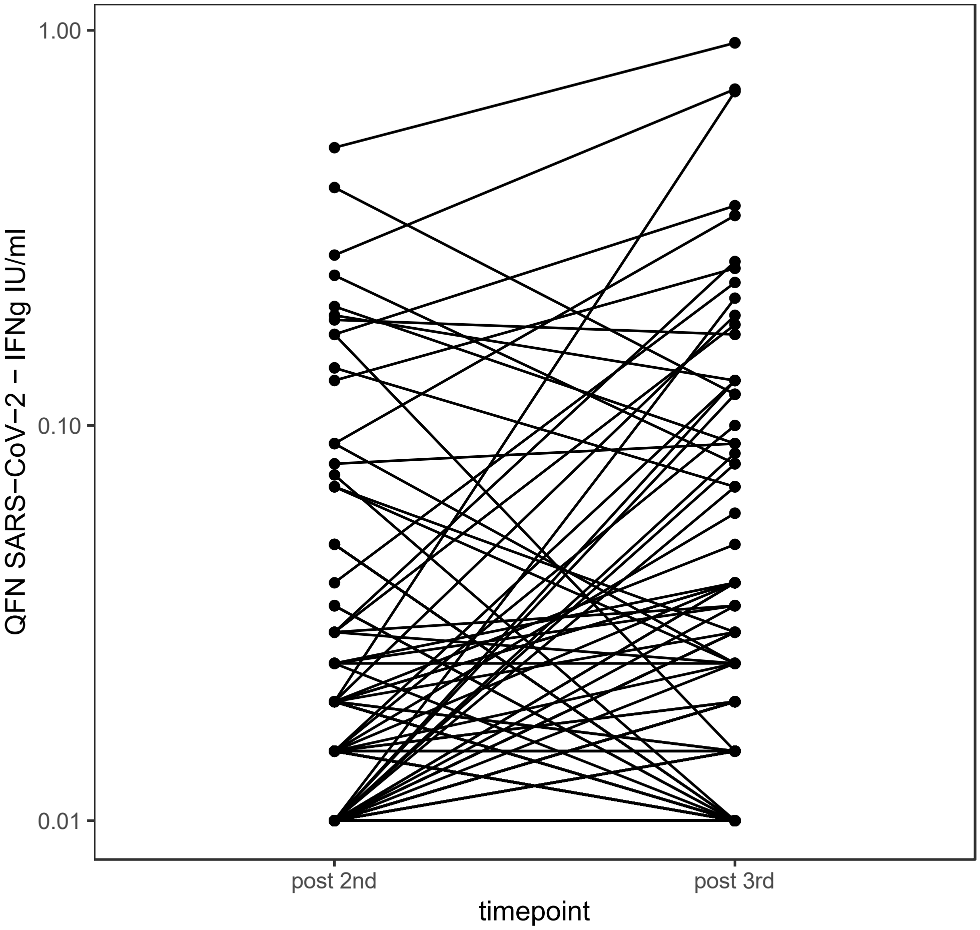

© 2021 Reindl-Schwaighofer R et al. *JAMA Intern Med.*

Supplemental eFigure 3. Reactogenicity following third vaccination. Severity of local and systemic side effects were graded by patients on a visual analogue scale (0: no symptoms, 1-2: mild, 3-4 moderate and 5 severe). There was no statistically significant difference between mRNA and vector group except for local pain at the injection side.

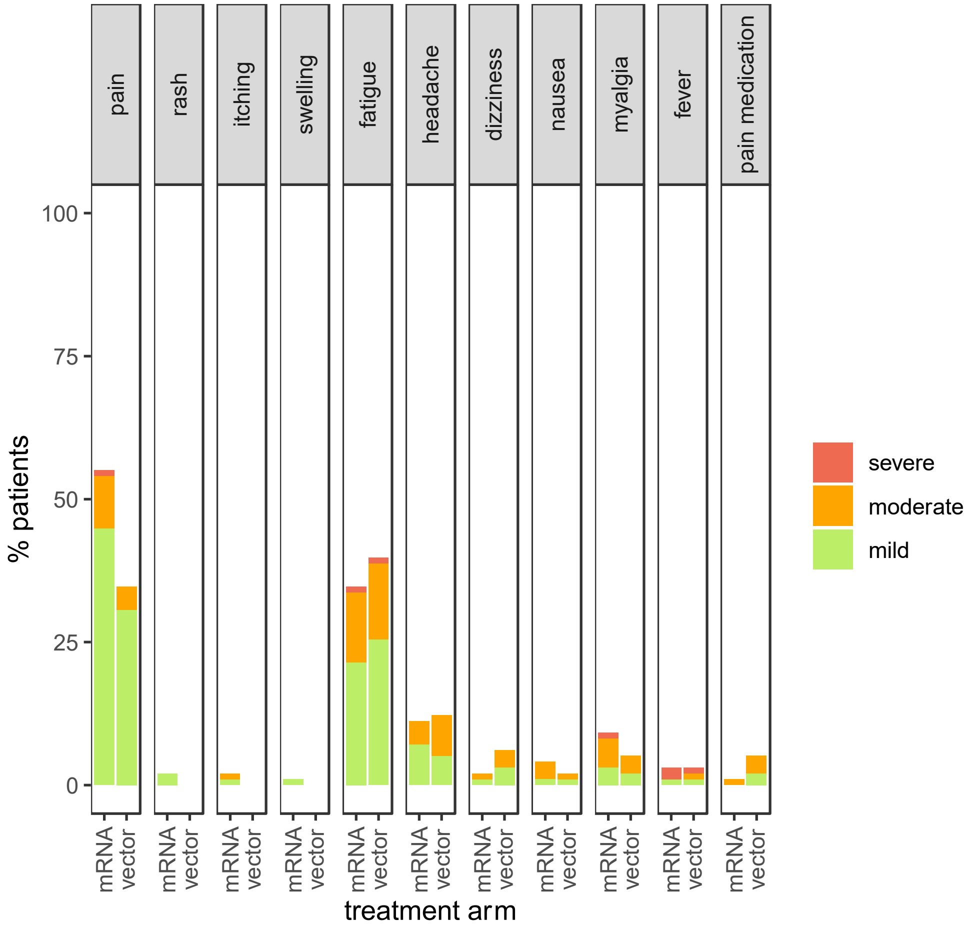

© 2021 Reindl-Schwaighofer R et al. *JAMA Intern Med.*

Supplemental eFigure 4. Interferon-gamma release assay results in healthy subjects after two doses of mRNA vaccine. All individuals had a response above 0.1 IU/ml (cut-off was calculated as mean plus two standard deviations in pre-pandemic control samples)

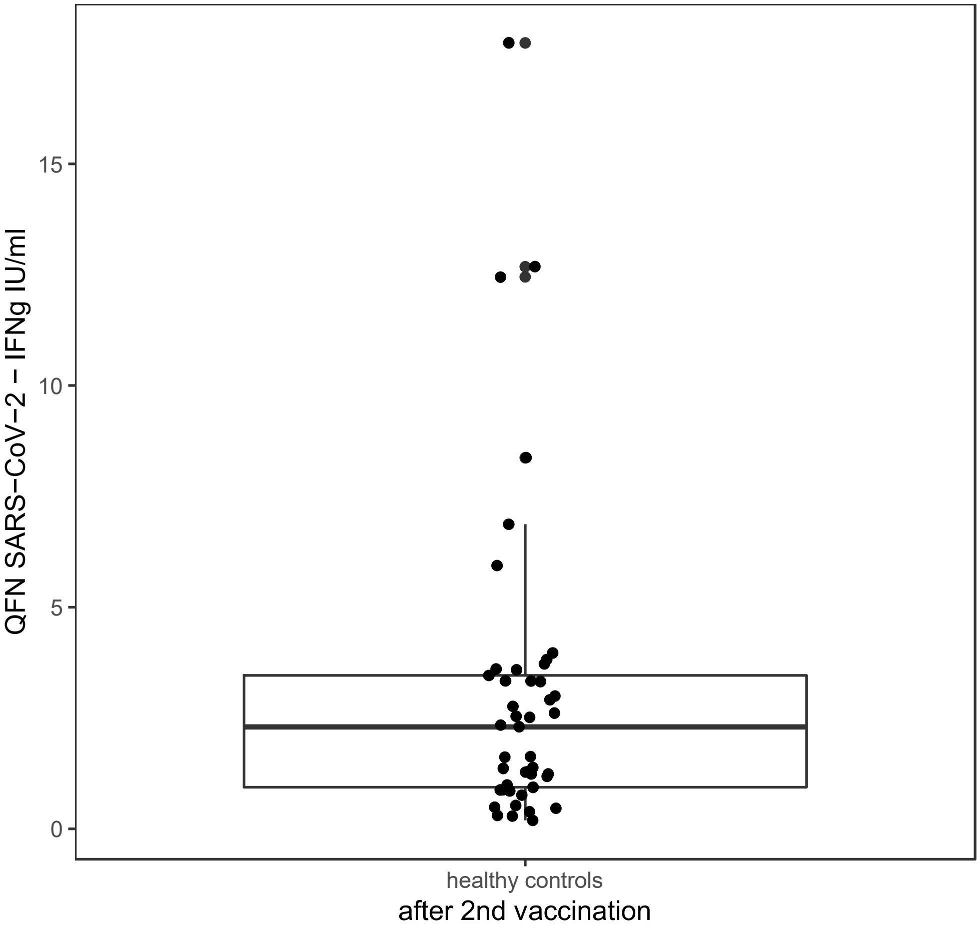

© 2021 Reindl-Schwaighofer R et al. *JAMA Intern Med.*
