# Supplementary material for: Comparison of SARS-CoV-2 Antibody Response 4 Weeks After Homologous vs Heterologous Third Vaccine Dose in Kidney Transplant Recipients: A Randomized Clinical Trial
Source: JAMA Intern Med. 2021 Dec 20;182(2):1–8. doi: 10.1001/jamainternmed.2021.7372 (PMC8689434; doi:10.1001/jamainternmed.2021.7372)
Supplement: Supplement 3. — Data Sharing Statement. [file jamainternmed-e217372-s003.pdf]

Data Sharing Statement

Reindl-Schwaighofer. Comparison of SARS-CoV-2 Antibody Response 4 Weeks After Homologous vs Heterologous Third Vaccine Dose in Kidney Transplant Recipients. *JAMA Intern Med*. Published December 20, 2021. doi:10.1001/jamainternmed.2021.7372

Data
Data available: Yes
Data types: Deidentified participant data
How to access data: roman.reindl-schwaighofer@meduniwien.ac.at
When available: With publication

Supporting Documents
Document types: None

Additional Information
Who can access the data: researchers whose proposed use of the data has been approved
Types of analyses: for a specified purpose (analysis of SARS-CoV-2 vaccine response in SOT)
Mechanisms of data availability: after approval of a proposal, additional IRB approval (if necessary) and signing of a data access agreement
